# Quantitative Proteomics Reveals the Dynamic Pathophysiology Across Different Stages in a Rat Model of Severe Traumatic Brain Injury

**DOI:** 10.3389/fnmol.2021.785938

**Published:** 2022-01-25

**Authors:** Weikang Luo, Zhaoyu Yang, Wei Zhang, Dan Zhou, Xiaohang Guo, Shunshun Wang, Feng He, Yang Wang

**Affiliations:** ^1^Department of Integrated Chinese and Western Medicine, Institute of Integrative Medicine, Xiangya Hospital, Central South University, Changsha, China; ^2^National Clinical Research Center for Geriatric Disorders, Xiangya Hospital, Central South University, Changsha, China; ^3^The College of Integrated Traditional Chinese and Western Medicine, Hunan University of Chinese Medicine, Changsha, China; ^4^Periodical Office, Hunan University of Chinese Medicine, Changsha, China; ^5^Medical School, Hunan University of Chinese Medicine, Changsha, China; ^6^Postpartum Health Care Department, Hunan Provincial Maternal and Child Health Care Hospital, Changsha, China; ^7^Department of General Surgery, Xiangya Hospital, Central South University, Changsha, China

**Keywords:** quantitative proteomic, severe traumatic brain injury, characteristic mechanisms, acute phase, subacute phase, tandem mass tag-based

## Abstract

**Background:**

Severe traumatic brain injury (TBI) has become a global health problem and causes a vast worldwide societal burden. However, distinct mechanisms between acute and subacute stages have not been systemically revealed. The present study aimed to identify differentially expressed proteins in severe TBI from the acute to subacute phase.

**Methods:**

Sixty Sprague Dawley (SD) rats were randomly divided into sham surgery and model groups. The severe TBI models were induced by the controlled cortical impact (CCI) method. We evaluated the neurological deficits through the modified neurological severity score (NSS). Meanwhile, H&E staining and immunofluorescence were performed to assess the injured brain tissues. The protein expressions of the hippocampus on the wounded side of CCI groups and the same side of Sham groups were analyzed by the tandem mass tag-based (TMT) quantitative proteomics on the third and fourteenth days. Then, using the gene ontology (GO), Kyoto encyclopedia of genes and genomes (KEGG), and protein–protein interaction (PPI), the shared and stage-specific differentially expressed proteins (DEPs) were screened, analyzed, and visualized. Eventually, target proteins were further verified by Western blotting (WB).

**Results:**

In the severe TBI, the neurological deficits always exist from the acute stage to the subacute stage, and brain parenchyma was dramatically impaired in either period. Of the significant DEPs identified, 312 were unique to the acute phase, 76 were specific to the subacute phase, and 63 were shared in both. Of the 375 DEPs between Sham-a and CCI-a, 240 and 135 proteins were up-regulated and down-regulated, respectively. Of 139 DEPs, 84 proteins were upregulated, and 55 were downregulated in the Sham-s and CCI-s. Bioinformatics analysis revealed that the differential pathophysiology across both stages. One of the most critical shared pathways is the complement and coagulation cascades. Notably, three pathways associated with gastric acid secretion, insulin secretion, and thyroid hormone synthesis were only enriched in the acute phase. Amyotrophic lateral sclerosis (ALS) was significantly enriched in the subacute stage. WB experiments confirmed the reliability of the TMT quantitative proteomics results.

**Conclusion:**

Our findings highlight the same and different pathological processes in the acute and subacute phases of severe TBI at the proteomic level. The results of potential protein biomarkers might facilitate the design of novel strategies to treat TBI.

## Introduction

Traumatic brain injury (TBI) is a significant cause of death and disability (Orešič et al., 2016). More than 50 million people suffer from TBI annually (Bazarian et al., 2018). It will be one of the top three specific neurological diseases by 2030 (World Health Organization [WHO], 2006; Maas et al., 2017). The pathological mechanisms of TBI consist of primary injury and secondary injury (Maas et al., 2017). About 10% of TBI patients are considered severe, and the risk of secondary damage increases with severity (Ruet et al., 2021). The process is complex and variable. First, glutamate-driven excitotoxic effects, oxidative stress, inflammatory reaction, ion imbalance, and metabolic disarray are significant pathological changes that induce neuronal loss (Rosenfeld et al., 2012; Maas et al., 2017). Second, excessive calcium influx will destroy the integrity of mitochondria, thereby depleting the energy source for cells (Maas et al., 2017). Third, the increased permeability of the blood-brain barrier (BBB) and lactic acidosis can lead to cerebral edema and intracranial hypertension (Stocchetti and Maas, 2014; Maas et al., 2017). Despite decades of effort to clarify the pathophysiology of TBI, the complex pathological mechanisms and biomarkers remain incompletely understood (Stocchetti and Maas, 2014; Irvine and Clark, 2018).

A series of fundamental researches on TBI have yielded promising results, but most clinical trials have failed (Maas et al., 2017; Xu et al., 2017; Irvine and Clark, 2018). The complex mechanisms can induce distinct pathologic changes in different stages. Therefore, drugs targeting a particular pathological process may have unique effects regarding the treatment periods (Diaz-Arrastia et al., 2014; Mohamadpour et al., 2019). For instance, NMDAR antagonist was reported to improve recovery of neurobehavioral and cognitive functions at the early stage of TBI. However, an NMDAR antagonist was not recommended because of the loss of functional NMDAR in the subacute period (Shohami and Biegon, 2014). It was futile when misusing NMDAR antagonists in the subacute stage of TBI (Shohami and Biegon, 2014). Regrettably, clinical protocols do not fully consider stage-specific pathology (Quaglio et al., 2017). Little is known about whether the outcomes change over time during the severe TBI (Beck et al., 2018). Thus, yielding insight into the pathology dynamics after TBI may aid in defining the time windows for the use of drugs.

Traumatic brain injury can trigger various proteins to change, closely related to the complex and dynamic pathophysiology (Yokobori et al., 2013; Song et al., 2019). Proteomics measures the systemic protein changes after TBI, reflecting the disease process. Currently, many studies use rat brain tissue for proteomic analysis. For instance, Liang et al. (2019) discovered that peripherin and calsenilin showed good potential as rat diffuse axonal injury biomarkers by proteomics, western blotting, and IHC analysis. In another study, Chen et al. (2018) discovered that proteomic profiling of mice brains exposed to blast-induced mild TBI revealed changes in axonal proteins and phosphorylated Tau. To rule out differences in species origin, human samples have been applied in studies of TBI proteomics. By comparing the molecular pattern of cortex in focal and diffuse TBI, some specific proteomic biomarkers of diffuse TBI were obtained, including peptides related to neurodegeneration (Tau and Fascin) and antioxidant defense (Glutathione *S*-transferase Mu 3, Peroxiredoxin-6, Thioredoxin-dependent peroxide reductase) (Abu Hamdeh et al., 2018). To harvest tissues conveniently, serum (Anada et al., 2018; Huie et al., 2019; Lindblad et al., 2021), cerebrospinal fluid (Lindblad et al., 2021), plasma (Bao et al., 2018; Ojo et al., 2018), and exosomal samples (Moyron et al., 2017) have been attempted for proteomics analysis. Thus, the yielded biomarkers in TBI were screened and verified from the differential proteins produced after comparison with various control groups. However, significant individual differences may exist because of the low number of human tissues. Screening of biomarkers of severe TBI is still in an exploratory phase. An in-depth analysis of proteome is necessary for exploring the specific mechanisms and biomarkers of post-TBI. Omics technology has implications for studying the underlying mechanisms at different periods. Sifting the differentially expressed proteins (DEPs) in the TBI acute and subacute phases through omics will provide clues for the pathogenesis and facilitate the development of novel target drugs in the future.

Quantitative proteomics, an accurate method to reveal DEPs in TBI, can simultaneously quantify multiple proteins and predict multiple targets and drug’s mechanism of action (Kou et al., 2018; Parker and Pratt, 2020; Suhre et al., 2021). Tandem mass tag-based proteomics (TMT) is a relatively quantitative proteomics technique that labels and analyzes multiple biological samples with high accuracy, sensitivity, and quality (Hughes et al., 2017). In combination with bioinformatics, TMT-based quantitative proteomics can effectively explore the complex biological process of disease (Mlecnik et al., 2018).

The hippocampus is an important region for the acquisition and consolidation of learning (Wu et al., 2013). Severe learning and memory deficits caused by TBI have been confirmed. Mechanical trauma occurs in the hippocampus as the initial primary injury and then causes secondary damage around the surrounding organizations (Lynch, 2004). In hippocampal tissue, changes in protein expression at different time points after TBI had been verified by proteomics (Cheng et al., 2018; Wang et al., 2021; Zhang et al., 2021). Wang et al. (2021) screened the potential biomarkers of acute-phase TBI in rats. Nevertheless, this study did not distinguish DEPs of the acute phase from DEPs of the subacute phase. Another proteomic profiling suggested that transthyretin (Ttr) can be up-regulated in the acute phase of TBI and supported thyroxine as a potential treatment (Zhang et al., 2021). However, it did not clarify the expression of Ttr during the subacute stage. Collectively, a comprehensive direct comparison of differential mechanisms at different stages of TBI is still lacking.

**FIGURE 1:**
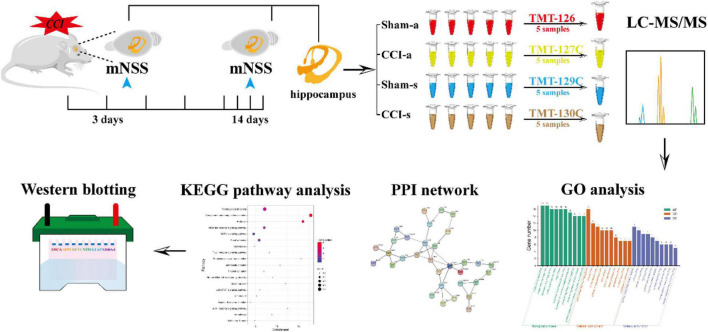
The schematic flowchart of the study. Rats were randomly divided into sham and CCI groups. The mNSS was evaluated at 3 and 14 days. The hippocampal tissues of brains at 3 and 14 days were collected for proteomics. Data were processed with LC-MS/MS and analyzed by bioinformatics. Finally, WB further validated the results of proteomics.

This study acquired the DEPs in the acute and subacute phases of severe TBI by TMT. The DEPs were analyzed by the Gene Ontology (GO) function, Kyoto encyclopedia of genes and genomes (KEGG), and protein-protein interaction (PPI), followed by functional analysis. Finally, further validation was achieved by Western blotting (WB). Our research aims to compare the DEPs between the acute phase and subacute stages to provide more insight into the potential pathogenesis at the protein level (Figure 1).

## Materials and Methods

### Animal Experiments

#### Preparation

Specific pathogen-free Sprague-Dawley (SD) rats (male, 230 ± 10 g) were purchased from Hunan Slake Jingda Laboratory Animal Co., Ltd. Research strictly adhered to the compliance of the Laboratory Animal Center of Central South University (CSU), and all animal experiments were approved by the Central South University Animal Ethics Committee (approval number: 201603112). All rats were housed in an aseptic environment (room temperature: 23°C, indoor relative humidity: 50%). Three rats per cage were housed during a 12 h light-dark cycle.

#### Model Building

As previously described, the Controlled Cortical Impact (CCI) model was performed (Zhou D. et al., 2020). Briefly, animals were anesthetized by intraperitoneal injections of 3% pentobarbital sodium (50 mg/kg) and immobilized. The scalp was cut along the midline. Then, the right skull was exposed. We used a portable drill bit to open a 5.0 mm skull window in the right parietal cortex. The center coordinates relative to the anterior fontanel: 1 mm posterior, 1 mm lateral. An electronically controlled pneumatic impact device was used to strike exposed dura at 5.0 mm in depth (5.0 mm from the cortical surface), impacting velocity at 6.0 m/s and retention time at 50 ms. Our CCI impact parameters are similar to severe injuries in previous studies and exceed the minimum depth (Clark et al., 1996; Campos-Pires et al., 2020).

#### Experimental Groups

Sixty SD rats were randomly divided into a sham-operated group of acute (Sham-a), acute group (CCI-a), sham-operated group of subacute (Sham-s), and subacute group (CCI-s). Each group had 15 rats. (i) Sham-a/Sham-s: CCI surgery but no damage to the cerebral cortex; (ii) CCI-a/CCI-s: CCI surgery and damage to the cerebral cortex. According to previous studies, 1–3 days post-TBI were regarded as the acute stage and 14 days later as the subacute stage (Izzy et al., 2019; Ruppert et al., 2020; O’Brien et al., 2021; Reed et al., 2021). Therefore, our study took 3 days post-TBI as the representative of the acute phase and 14 days as the subacute phase.

#### Modified Neurological Severity Score

After completing the model, the sham-a/CCI-a and Sham-s/CCI-s groups of rats were evaluated for the modified neurological severity score (mNSS) by the single-blind method on days 3 and 14, respectively. The mNSS comprises sensory, motor, balance, and reflex tests (Chen et al., 2001; Zhou D. et al., 2020). The score ranges from 0 to 18, as follows: motor (6 points), sensory (2 points), beam balance (6 points), reflexes absent, and abnormal movements (4 points). It was divided into three categories: 1–6 as mild brain damage, 7–12 as moderate brain damage, 13–18 as severe brain damage. One score point is awarded for the inability to perform the test or for the lack of a tested reflex in injury severity scores. The higher the score, the more severe the injury is (Chen et al., 2001).

#### Hippocampal Tissue Collection

Considering that the cortical impact can affect both the ipsilateral and contralateral side of the brain tissues (Lowenstein et al., 1992), we only obtained the hippocampus on the injured side of CCI groups and the hippocampus on the same side of Sham groups. Brain samples in the injured side from the rats on days 3 and 14 after TBI, respectively. All rats were anesthetized with 3% pentobarbital sodium by intraperitoneal injection (50 mg/kg). 0.9% normal saline was used for systemic perfusion after anesthesia. Then, the brain tissues were removed quickly. Finally, the hippocampal tissues were separated and stored at –80°C until analysis.

### Tandem Mass Tag-Based Quantitative Proteomics

#### Preparation of Protein Samples

Five samples in the same group were mixed into one for detection. First, 1000 μL of working fluid (25 mM Tris•HCI pH 7.6, 150 mM NaCl, 1% NP-40, 1% sodium deoxycholate, 1% SDS) was added into samples. Second, the mixture was sonicated in ice for 5 min and centrifuged at 14000 r/min for 15 min at 4°C. Finally, protein concentration was measured with a BCA protein quantification kit (Thermo Scientific, United States).

#### Tandem Mass Tag-Based Labeling

Each sample was reduced to 100 μg supernatant and subjected to alkylation and acetone precipitation. After suspension, the sample was digested with trypsin, mixed thoroughly, centrifuged briefly, and incubated overnight at 37°C with shaking. Next, the mixture was centrifuged at high speed for 10 min and transferred to a new ep tube. Pre-equilibrated TMT to room temperature before opening. Then, 41 μL of anhydrous acetonitrile (anhydrous ACN) was added, mixed thoroughly, and centrifuged to collect the TMT solution. Next, 20 μL of TMT solution was pipetted into the corresponding sample, mixed, and centrifuged. After a 1-h incubation at room temperature, 100 mM hydroxylamine was added and incubated for 15 min to stop the reaction. Polypeptide samples were Sham-a (TMT-126), CCI-a (TMT-127C), Sham-s (TMT-129C), CCI-s (TMT-130C). TMT labeling was carried out by the manufacturer’s instructions (Thermal Science, United States). Sodium deoxycholate was cleaned with 2% TFA after TMT labeling.

#### High pH Reverse Phase Fractionation

Polypeptide samples (100 μg) were carried out by High pH reverse phase fractionation (pH = 10). Chromatographic column: XBridge BEH C 18 XP Column (150 × 2.1 mm, Waters). The mobile phase A was ammonium formate (AF) water solution: 10 mM, pH = 10. Mobile phase B: 10 mM AF, 10% H_2_O, 90% ACN, pH = 10. Samples were divided into 120 min gradient intervals. Gradient B: 5–28% for 78 min, 28–50% for 12 min, 50–80% for 2 min, 80% for 4 min, 80–5% for 2 min, 5% for 20 min. The polypeptide was separated into 180 parts and collected in 40 s intervals. Lastly, all samples were merged into 20 components and then were vacuum dried and stored at –80°C before liquid chromatography-mass spectrometry (LC-MS)/MS analysis.

#### Liquid Chromatography-Mass Spectrometry/Mass Spectrometry Analysis

Firstly, 1 μg of the peptide was isolated and analyzed by nano UPLC (EASY-nLC1200) and Q Exactive mass spectrometry (Thermo Finnigan). Each component was replicated three times. Separation of peptides was performed by reverse-phase column (100 μm, ID × 15 cm, Reprosil-Pur120 C18-AQ, 1.9 μm) in buffer A (0.1% FA, 2% ACN) and buffer B (0.1% FA, 80% ACN) with a flow rate of 300 nL/min. Linear gradient: 6–28% for 70 min, 28–40% for 12 min, 40–100% for 2 min, 100% for 2 min, 100–2% for 2 min, 2% for 2 min. Next, the mass spectrometry detection was performed under positive ion mode by Q Exactive mass spectrometry (90 min/sample). Data were acquired in DDA mode (20 pieces each time). The standardized collision energy was set to 32%. The isolation window was set to 2 m/z. The dynamic exclusion time was set to 30 s. MS parameters were as follows: (1) Primary mass spectrometry: Scanning range of the parent ions was 350–1600 m/z; Resolution of 70,000 at 200 m/z; The automatic gain control (AGC) target was set to 3E6; Maximum ion injection time (Max IT) was set to 50 ms; (2) Secondary mass spectrometry: Resolution of 17,500 at 200 m/z; AGC target was set to 1E5; Max IT was set to 100 ms.

#### Quantitative Data Processing of Tandem Mass Tag-Based

Raw MS data were processed with MaxQuant software (version 1.5.6.0) for data search and quantitative analysis. The protein database was UNIPROT_RAT_2016_09. The false discovery rate (FDR) was less than 1% at both peptide and protein levels.

### Screening of Differentially Expressed Proteins

The screening condition of DEPs were as follows: unique peptides ≧ 2; *p*-value < 0.05; fold change > 1.2 (up-regulation) or <0.83 (down-regulation).

### Bioinformatics Analysis

Gene ontology (GO) and KEGG analysis were conducted using Metascape^1^. *P*-values were calculated based on the accumulative hypergeometric distribution according to the Metascape. The protein--protein interaction (PPI) network was constructed using the STRING database (version 11.0)^2^.

### Western Blotting

#### Sham-a/CCI-a Groups

The hippocampal tissues were homogenized in 500 μL ice-cold lysates. After centrifugation (12,000 r/min, 4°C, 10 min), protein concentrations were determined by the BCA protein measurement kit (Thermo Scientific, United States). Proteins were separated on 10% polyacrylamide electrophoresis gels (SDS-PAGE), then transferred onto PVDF membranes. Membranes were blocked with 5% skim milk powder in 1 × PBST (20 mM Tris-HCl, 150 mM NaCl, 0.05% Tween 20) for 1 h, followed by incubation with rabbit-anti SPP1 (22952-1-AP, 1:3,000, Proteintech, United States), rabbit-anti SNCA (10842-1-AP, 1:1,000, Proteintech, United States), rabbit-anti SYN1 (20258-1-AP, 1:5,000, Proteintech, United States) and rabbit-anti GAPDH (10494-1-AP, 1:5,000, Proteintech, United States) at 4°C overnight. Membranes were washed with 1 × PBST three times for 15 min each after incubated. Then, membranes were incubated with horseradish peroxidase (HRP)-conjugated IgG of goat anti-rabbit (SA00001-2, 1:6,000, Proteintech, United States) for 90 min at room temperature, and washed with 1 × PBST three times for 15 min each. Subsequently, blots were detected by the ECL reagent (Thermo Scientific, United States). The protein bands were quantified using Quantity one v 4.6.2 software finally. GAPDH was used as a reference for relative expression.

#### Sham-s/CCI-s Groups

Primary antibodies changed to rabbit-anti VIM (10366-1-AP, 1:4,000, Proteintech, United States), mouse-anti GFAP (60190-1-Ig, 1:10,000, Proteintech, United States), rabbit-anti S100A4 (ab197896, 1:1,000, Proteintech, United States), and rabbit-anti GAPDH (10494-1-AP, 1:5,000, Proteintech, United States). After overnight incubation, membranes were washed with 1 × PBST three times for 15 min each. Then, membranes were incubated with horseradish peroxidase (HRP)-conjugated IgG of goat anti-mouse (SA00001-1, 1:5,000, Proteintech, United States) and IgG of goat anti-rabbit (SA00001-2, 1:6,000, Proteintech, United States) for 90 min at room temperature. The remaining steps were the same as “Sham-a/CCI-a Groups”. All data were quantified by Quantity One software.

### Immunofluorescence

The paraffin-embedded brain was cut into sections (thickness 3 μm). First, brain sections were deparaffinized in xylene and rehydrated by ethanol (2 × xylene, 100% ethanol, 100% ethanol, 95% ethanol, 75% ethanol, each 5 min). Then, sections were repaired using 800 mL citrate buffer antigen for 20min and washed 3 × 5 min with 0.1 M phosphate-buffered saline (PBS). After being blocked with blocking solution for 120 min, tissues were incubated with the primary antibodies overnight at 4°C: anti-GFAP (1:1500, Sigma, Millipore-MAB360) and anti-IBA-1 (1:400, WAKO, 019-19741). After washing three times (10 min per time) with 0.1 M PBS, slides were incubated with secondary antibodies for 60 min: anti-mouse CY3 (1:1000, Jackson Immunoresearch, United States) for GFAP detection and anti-rabbit Alexa flour 488 (1:1000, Jackson Immunoresearch, United States) for IBA-1 detection. Subsequently, sections were re-washed in 0.1 M PBS for three times (5 min per time). Finally, all sections were counterstained with DAPI (1:100, Solarbio) and mounted using glycerin.

### H&E Staining

The paraffin sections of rat brain tissues (5 μm) were deparaffinized by xylene and ethanol (2 × xylene, 100% ethanol, 100% ethanol, 95% ethanol, 75% ethanol, each 5 min), and stained with hematoxylin-eosin (Servicebio, China) method (hematoxylin 4 min, eosin 20 s). Then, sections were dehydrated in ethanol and xylene and sealed with synthetic resin.

### Statistical Analysis

Statistical analysis was performed with SPSS 26.0. The normality of data was tested by the Shapiro–Wilk normality test. Data were analyzed by independent *t*-tests and presented as the mean ± standard deviation when normality was passed. The *p* < 0.05 were considered statistically significant.

## Results

### Neurological Deficits in Traumatic Brain Injury Models

**FIGURE 2:**
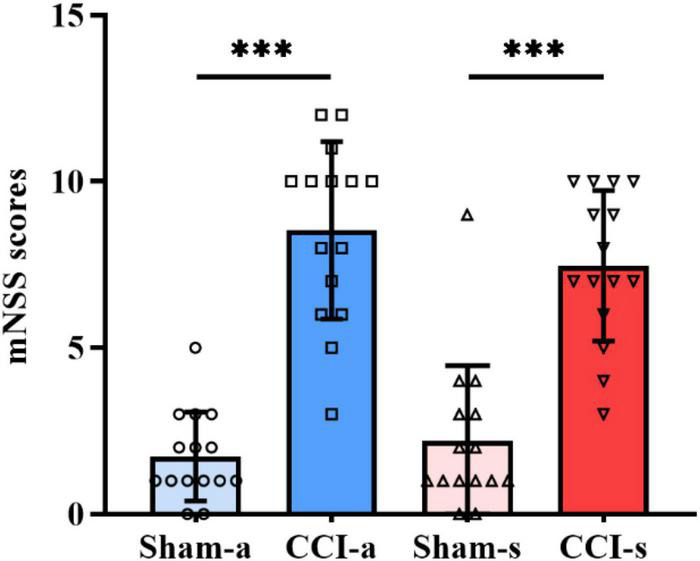
Neurobehavioral scores on days 3 and 14 of TBI. The mNSS of Sham-a and CCI-a groups on day 3 of TBI (left two groups). The mNSS of Sham-s and CCI-s groups on day 14 of TBI (right two groups). *N* = 15; Data were expressed as mean ± SD; ***p < 0.001.

To evaluate the neurological deficits of rats at the acute phase of severe TBI, mNSS was performed on the 3rd day. The scores of the CCI-a group (8.53 ± 2.669) were significantly higher than Sham-a (*p* < 0.001), as shown in Figure 2. Similarly, mNSS of the Sham-s group was considerably lower than that of CCI-s (7.47 ± 2.264) on day 14 (*p* < 0.001) (Figure 2). These results indicated that the severe TBI models were established successfully. In addition, the mNSS showed a downward trend from the acute stage to the subacute phase.

### Histological Analysis of Traumatic Brain Injury Models

**FIGURE 3:**
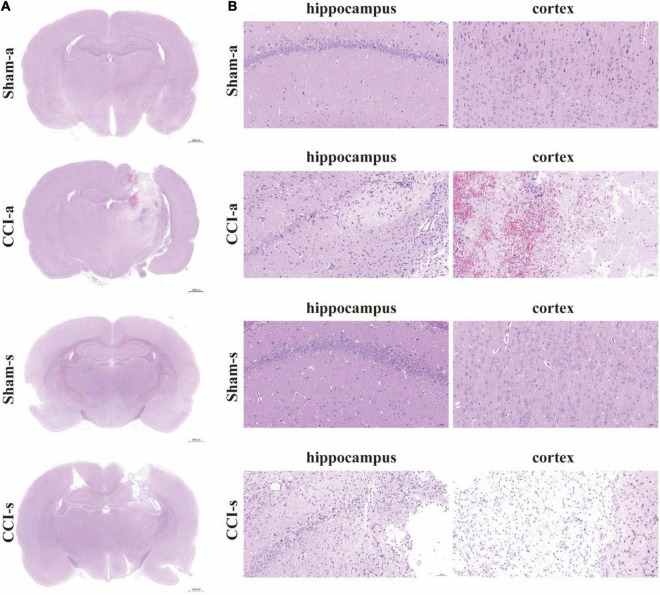
H&E staining of brain tissues. **(A)** The brain sections in different groups were stained with H&E and examined by high magnification. **(B)** H&E staining of cortex and hippocampus of the injured side. Sham-a, a sham-operated group of acute, CCI-a, CCI-operated group of acute, Sham-s, a sham-operated group of subacute; CCI-s, CCI-operated group of subacute group.

**FIGURE 4:**
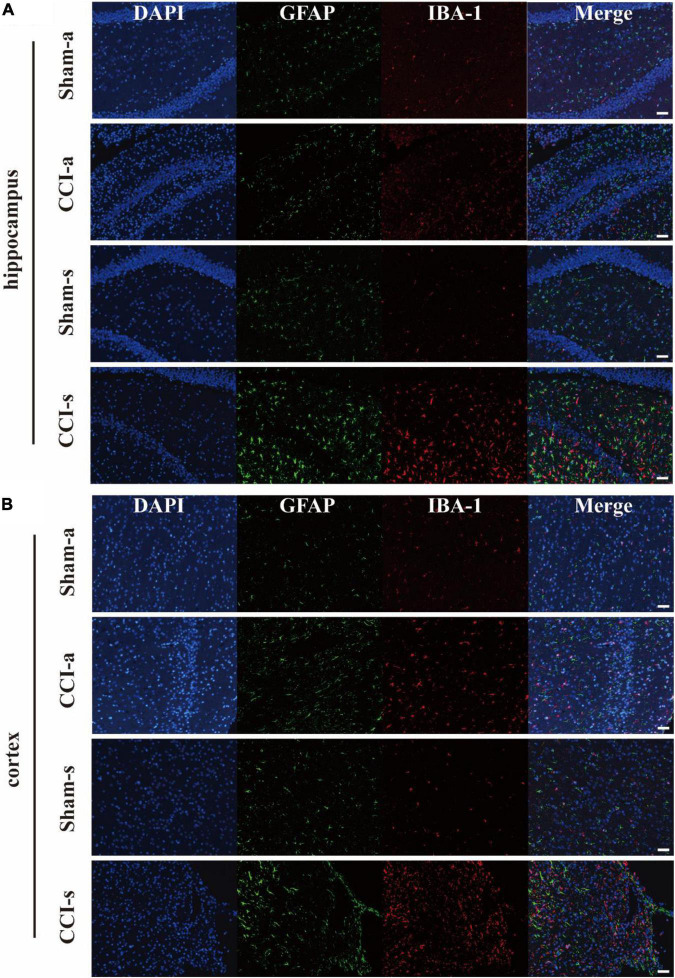
The activation of astrocyte and microglia after severe TBI. GFAP antibody and IBA-1 antibody are specific for astrocytes and microglia, respectively. **(A)** Representative immunofluorescent staining for GFAP (Green) and IBA-1 (Red) in the injured hippocampus. **(B)** Representative immunofluorescent staining for GFAP (Green) and IBA-1 (Red) in the injured cortex. Scale bar = 50 μm.

The brain injury was assessed by the H&E staining. Under low magnification, we found that the brain parenchyma of TBI models was dramatically impaired (Figure 3A). The high magnification illustrated neuronal necrosis enlarged intercellular spaces and immune cell infiltration in the CCI-a and CCI-s groups (Figure 3B). GFAP and IBA-1 antibodies are specific for astrocytes and microglia, respectively. Thus, evaluation of neuroinflammation occurring after severe TBI was obtained using GFAP and IBA-1 antibodies by immunofluorescence. The regions we examined selected hippocampus and cortex on the injured. Overall, the expression and activation of microglia and astrocytes were elevated in the CCI groups relative to Sham groups (either 3 or 14 days) (Figure 4).

### Differentially Expressed Proteins Analysis

**FIGURE 5:**
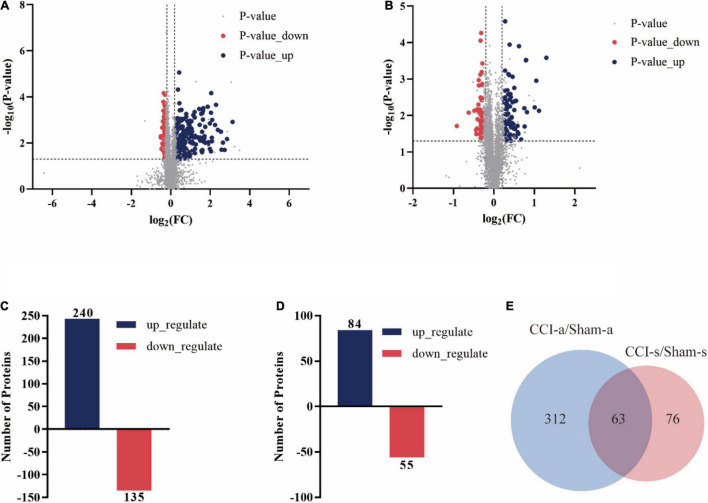
Differentially expressed proteins of CCI rats in the hippocampal tissues on days 3 and 14 of TBI. The volcano plot showed the DEPs, up-regulated (blue) and down-regulated (red) between Sham-a/CCI-a groups **(A)** and Sham-s/CCI-s **(B)** groups of 5535 proteins: unique peptides ≧ 2; *p*-value < 0.05; FC > 1.2 or < 0.83. **(C)** DEPs in the Sham-a/CCI-a groups (240 up-regulated; 135 down-regulated). **(D)** DEPs in the Sham-s/CCI-s groups (84 up-regulated; 55 down-regulated). **(E)** Venn diagram of stage-specific DEPs and the shared DEPs of acute and recovery phases of TBI.

We identified a total of 6,591 proteins, of which 5,535 were quantifiable. Three-hundred and seventy-five proteins had gene names (240 up-regulated, 135 down-regulated) between the Sham-a and CCI-a groups (Figures 5A,C). Besides, 139 DEPs had gene names in the Sham-s and CCI-s groups, including 84 up-regulated proteins and 55 down-regulated proteins, as shown in Figures 5B,D. To identify the exclusive DEPs of different periods, we excluded 63 overlapping DEPs by Venn diagram (Figure 5E). Then, 312 DEPs were detected in the CCI-a/Sham-a group and 76 in the CCI-s/Sham-s. These results indirectly proved the heterogeneous condition between the acute and recovery stages.

### Gene Ontology Analysis for Differentially Expressed Proteins

Gene ontology analysis was performed to obtain the biological functions for the 312 exclusive DEPs in the acute stage, 76 exclusive DEPs in the subacute phase, and 63 shared DEPs.

#### Acute Phase

**FIGURE 6:**
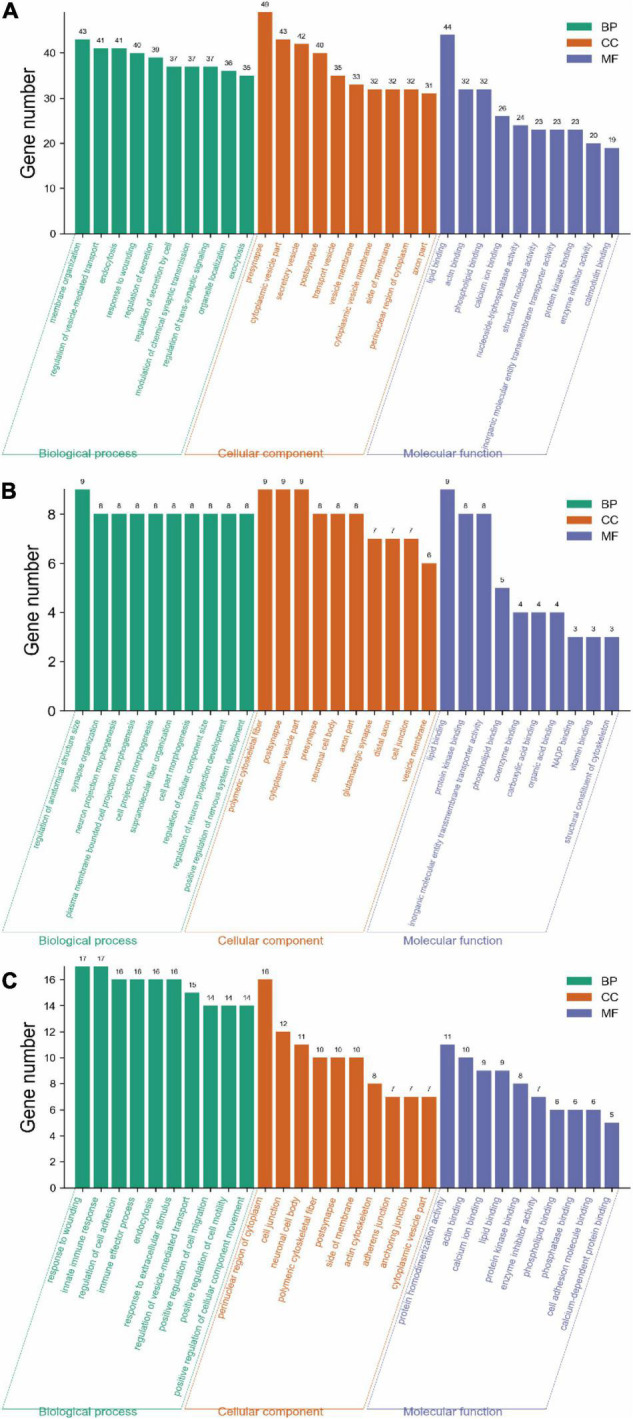
Gene ontology analysis of DEPs on days 3 and 14 of TBI. **(A)** GO annotation of 312 DEPs in the Sham-a/CCI-a groups (excluding 63 shared DEPs). **(B)** GO annotation of 76 DEPs in the Sham-s/CCI-s groups (excluding 63 shared DEPs). **(C)** GO annotation of 63 overlapping DEPs in the Sham-a/CCI-a and Sham-s/CCI-s groups.

For the biological process, the proteins were mainly involved in membrane organization (13.78%), regulation of vesicle-mediated transport (13.14%), endocytosis (13.14%), response to wounding (12.82%), actin regulation of secretion (12.50%), and regulation of secretion by cell (11.86%) (Figure 6A).

The top five cellular components were presynapse (15.71%), cytoplasmic vesicle part (13.78%), secretory vesicle (13.46%), postsynapse (12.82%), and transport vesicle (11.22%) (Figure 6A).

In molecular function, protein binding was the top lipid binding (14.10%) and followed by actin-binding (10.26%) and phospholipid binding (10.26%), as shown in Figure 6A.

#### Subacute Phase

Compared with the acute phase, the subacute stage was somewhat divergent. DEPs in the biological process was primarily included regulation of anatomical structure size (11.84%), synapse organization (10.53%), neuron projection morphogenesis (10.53%), and plasma membrane bounded cell projection morphogenesis (10.53%) (Figure 6B).

In the cellular component, DEPs were mainly located in the polymeric cytoskeletal fiber (11.84%) and followed by the postsynapse (11.84%) (Figure 6B).

For molecular function, DEPs were associated with lipid binding (11.84%), protein kinase binding (10.53%), and inorganic molecular entity transmembrane transporter activity (10.53%) (Figure 6B).

#### Shared Differentially Expressed Proteins Between Acute and Subacute Phases

The top five biological processes were a response to wounding (26.98%), innate immune response (26.98%), regulation of cell adhesion (25.40%), immune effector process (25.40%), and endocytosis (25.40%) (Figure 6C).

The cellular components included mainly the perinuclear region of cytoplasm (25.40%), cell junction (19.05%), neuronal cell body (17.46%), polymeric cytoskeletal fiber (15.87%), and postsynapse (15.87%) (Figure 6C).

For molecular function, the predominantly represented GO terms mainly included protein homodimerization activity (17.46%) and actin binding (15.87%) (Figure 6C).

### Kyoto Encyclopedia of Genes and Genomes Pathway Analysis for Differentially Expressed Proteins

**FIGURE 7:**
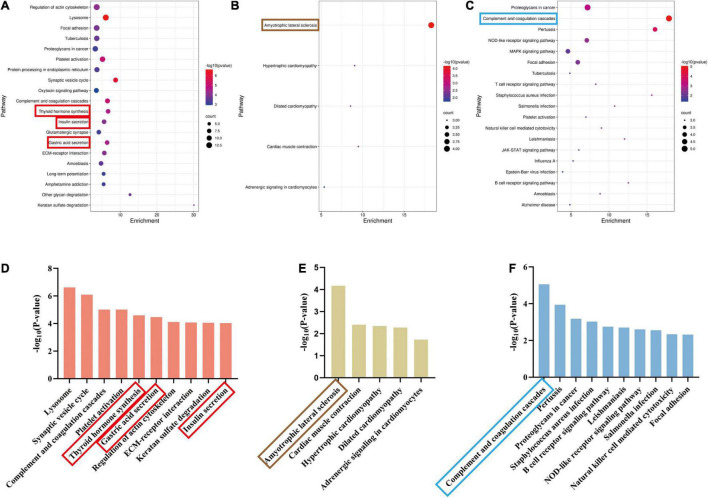
Significant pathways of acute and recovery stages of TBI. (*p* < 0.05). **(A)** The significant pathways in the Sham-a/CCI-a groups (excluding 63 shared DEPs). The ordinate represents pathways. **(B)** The significant pathways in the Sham-s/CCI-s groups (excluding 63 shared DEPs). The ordinate represents pathways. **(C)** The significant pathways of 63 shared DEPs. The ordinate represents pathways. **(D)** The top ten significant pathways in the Sham-a/CCI-a groups. **(E)** The top 10 significant pathways in the Sham-s/CCI-s groups. **(F)** The top 10 significant pathways of 63 shared DEPs. The red rectangle represents three endocrine-related pathways; brown rectangle, amyotrophic lateral sclerosis; blue rectangle, complement, and coagulation cascades.

We identified the related pathways through KEGG analysis based on the two groups of exclusive DEPs. In the acute stage, 64 pathways had a significant correlation (*p* < 0.05) (Figures 7A,D and Supplementary Table 1). Conversely, only five pathways were significantly enriched in the subacute stage (Figure 7B and Supplementary Table 2). For shared pathway analysis between the two stages, we screened the KEGG pathways based on 63 shared DEPs (Figures 7C,F and Supplementary Table 3). Both phases were mainly related to the complement and coagulation cascades pathway (Figure 7F).

Next, we ended up with ten unique pathways in the Sham-a/CCI-a group and five in the Sham-s/CCI-s after comparing the top ten pathways. The lysosome was the most dominant, followed by the synaptic vesicle cycle (Figure 7D). Nevertheless, amyotrophic lateral sclerosis (ALS) was most relevant in the subacute stage (Figure 7E). Excitingly, we obtained three endocrine-related pathways in the acute phase, including gastric acid secretion, insulin secretion, and thyroid hormone synthesis. Further analysis of upregulated and downregulated proteins revealed that the expression of Slc4a2 and Ezr were increased in the gastric acid secretion, and most proteins (Alb, Gpx1, Ttr, Slc5a5, Pdia4) were increased in the thyroid hormone pathway (Supplementary Table 4). However, decreased expression of all proteins related to insulin secretion (Camk2b, Prkcg, Snap25, Camk2a, Rab3a, Pclo, Stx1a, Adcy9) (Supplementary Table 4).

### Protein–Protein Interaction Analysis Was Conducted Using STRING Database

**FIGURE 8:**
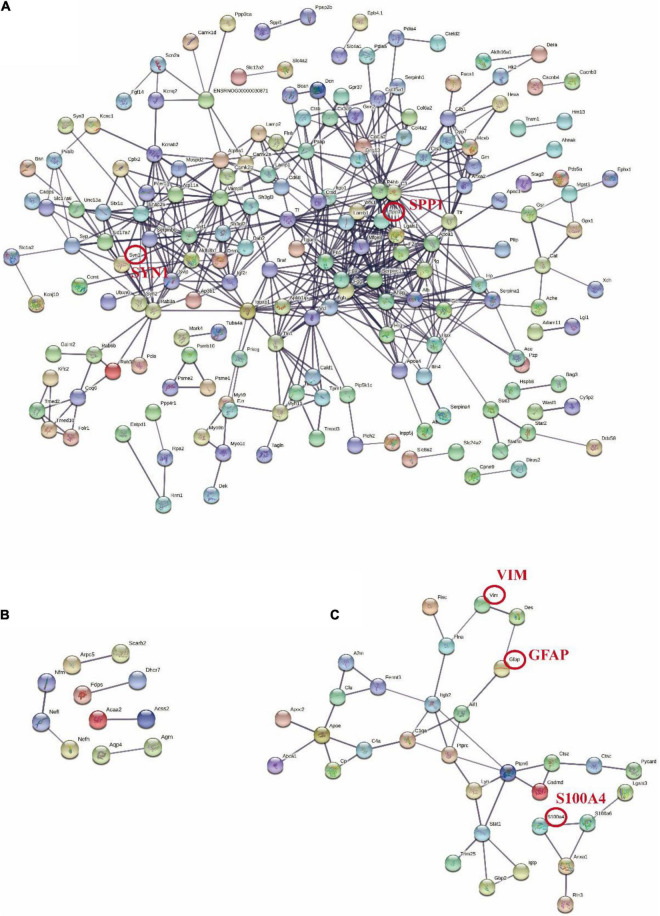
Protein-protein interaction networks of different stages of TBI constructed in STRING. One dot represents a protein. The pattern in the node represents the predicted three-dimensional structure. Each line represents the relationship between the proteins. The thickness of the line represents the correlation strength. (Minimum required interaction score: high confidence = 0.700). **(A)** PPI network of 312 DEPs in the Sham-a/CCI-a groups. **(B)** PPI network of 76 DEPs in the Sham-s/CCI-s groups. **(C)** PPI network of 63 shared DEPs. According to the FC order, six proteins were randomly selected from the top 20 up-regulated and down-regulated, respectively, for verification, and five were in the network (red circles).

The STRING database was performed to validate the interrelation among differential proteins. After DEPs were submitted to STRING, we set the high confidence at 0.700 and hid disconnected nodes in the network. As shown in Figure 8A, the network revealed a potential relationship between Spp1, Syn1, Fgb, Sperpinc1, and so on in the acute phase. Another network illustrated the possible relationship of 76 DEPs in the subacute stage (Figure 8B). From this plot, the number of DEPs varied extensively across diverse stages. These nodes might play a critical role at various phases of severe TBI, and the differences probably reflected the dynamic pathological processes. In addition, we also labeled the 63 shared DEPs in the PPI network, exhibiting a potential relationship between the acute and subacute phases (Figure 8C). These protein targets may guide further application with targeted drugs during the initiation and progression of TBI.

### Validation of Differentially Expressed Proteins by Western Blotting

**FIGURE 9:**
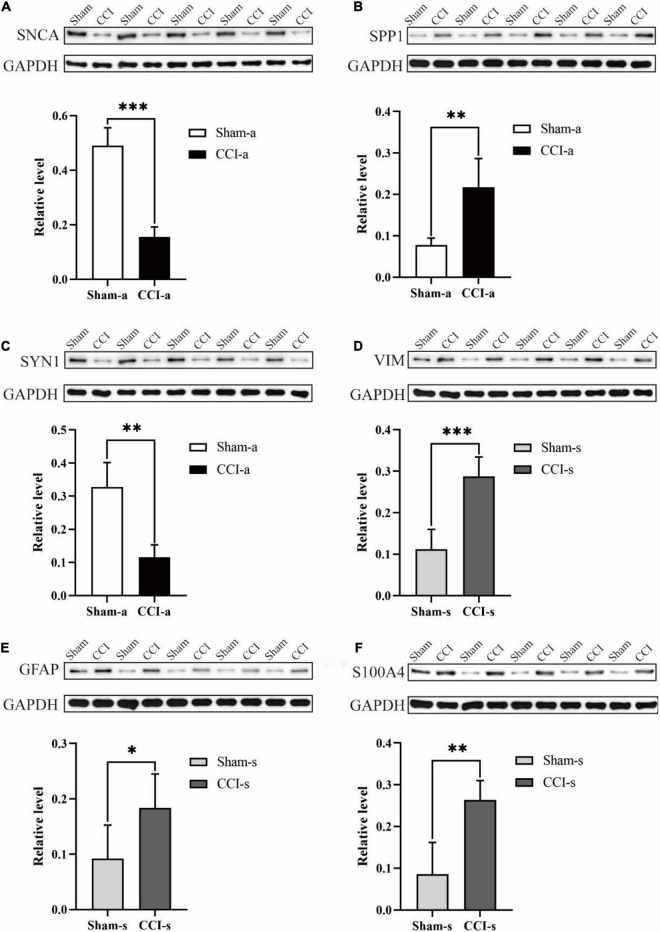
Validation of randomly selected DEPs (*N* = 5, data were expressed as mean ± SD. *p < 0.05; **p < 0.01; ***p < 0.001). We randomly selected SNCA, SPP1, and SYN1 in Sham-a/CCI-a groups and VIM, GFAP, and S100A4 in Sham-s/CCI-s groups to verify. These were randomly selected from the top 20 up-regulated and down-regulated proteins, respectively. **(A)** Protein expression and relative level of SNCA. The experiment was repeated five times with similar results. **(B)** Protein expression and relative level of SPP1. The experiment was repeated five times with similar results. **(C)** Protein expression and relative level of SYN1. The experiment was repeated five times with similar results. **(D)** Protein expression and relative level of VIM. The experiment was repeated five times with similar results. **(E)** Protein expression and relative level of GFAP. The experiment was repeated five times with similar results. **(F)** Protein expression and relative level of S100A4. The experiment was repeated five times with similar results.

To validate quantitative proteomics results, we randomly selected three of the top 20 proteins from 375 DEPs for WB (SNCA, SPP1, and SYN1). Compared with the Sham-a group, SNCA and SYN1 were decreased in the CCI-a (*p* = 0.000; *p* = 0.001; *n* = 5; Figures 9A,C). Conversely, SPP1 were increased significantly in the CCI-a group (*p* = 0.000; *n* = 5; Figure 9B). These results were matched to those of proteomics analysis (Supplementary Table 5). We next selected three proteins among the 139 DEPs to verify. The results were also in agreement with the quantitative proteomics. As shown in Figures 9D–F, TBI caused a remarkable increased of VIM, GFAP and S100A4 compared with the Sham-s (*p* = 0.000; *p* = 0.044; *p* = 0.002; *n* = 5). Overall, WB confirmed the reliability of quantitative proteomics (Supplementary Tables 5, 6).

## Discussion

This study demonstrated differential proteomics in the acute and subacute stages of severe TBI. Both were mainly related to the complement and coagulation cascades pathway. After excluding 63 shared DEPs, we separately screened the distinct pathways between the two phases. GO analysis indicated that they are involved in many biological processes. Consistent with previous findings, the acute stage emphasized platelet activation and nerve-related pathways (Schwarzmaier et al., 2010; Sandsmark et al., 2019; Xu et al., 2021). Nonetheless, we noted other unexpected endocrine-related pathways, including gastric acid secretion, thyroid hormone synthesis, and insulin secretion. The ALS pathway was only related to the subacute phase. Analysis of six proteins confirmed the reliability and accuracy of quantitative proteomics. This work was dedicated to distinguishing the differential pathways in various stages of severe TBI and added strategies for achieving precise therapy at the protein level.

Only very few studies point out that head injury can cause changes in stomach acid (Rachfalska et al., 2020). The increase in intracranial pressure caused by brain damage may affect different hypothalamic nucleus or brainstem areas, resulting in excessive stimulation of the vagus nerve or paralysis of the sympathetic nervous system, thereby enhancing gastric acid secretion (Lewis, 1973). However, the pathophysiological mechanism has remained mysterious (Rachfalska et al., 2020). Previous research indicated that Ezr knockdown could inhibit gastric acid secretion by expanding canalicular apical membranes in parietal cells (Tamura et al., 2005). Another study disclosed that the expression of Ezr is up-regulated in the injured hippocampus on days 4 and 14 after brain injury (Andersson et al., 2013). But the association between Ezr and gastric acid secretion remains to be determined during severe TBI. Our study showed that Ezr is highly expressed in the gastric acid secretion pathway. Given the above results and discussion, we conclude that Ezr may be a key molecule in increasing gastric acid secretion during the acute stage of severe TBI. These findings merit further validation using the samples of TBI patients.

Besides, two pathways, thyroid hormone synthesis and insulin secretion were also significantly enriched at the acute phase. Thyroid hormones can promote recovery and neuron regeneration after brain damage (Liu and Brent, 2018; Lin et al., 2020), and the brain thyroid hormone perturbation may be a potential pathogenetic factor in hippocampal sclerosis (Nelson et al., 2016). Ttr, a pivotal carrier of thyroid hormones, can cross the BBB and preferentially bind T4 (Richardson et al., 2015; Zhang et al., 2021). The latest research confirmed that Ttr is regarded as a novel therapeutic target in the acute stage of TBI (Zhang et al., 2021). However, whether it genuinely applies to the entire TBI stage is unknown. In our study, Ttr seems inappropriate for use as a molecular target in the subacute phase of severe TBI. We also found the upregulation of Alb, Gpx1, Slc5a5, and Pdia4 associated with thyroid hormone synthesis. Clarifying the appropriate stage of molecular therapy contributes to avoiding ineffective treatment due to changes in the pathological process of TBI. Apart from thyroid disorders, TBI also induces hyperglycemia (Kurtz and Rocha, 2020). The central infusion of IGF-1 was able to increase hippocampal neurogenesis and improve neurobehavioral function (Carlson and Saatman, 2018). The hippocampal tissue we test may be the reason for the enrichment of the above two pathways. All proteins associated with insulin secretion were down-regulated, which means insulin secretion was reduced in the acute stage of severe TBI. These proteins emerge as candidate therapeutic targets for hyperglycemia after severe TBI. Exogenous thyroid supplementation and early glycemic control may effectively treat complications in the acute phase of severe TBI.

Upon brain injury, hippocampal cells can release brain-derived microparticles, which are released into the circulating system through the incomplete BBB in a platelet-dependent manner, promoting intravascular coagulation and platelets activation (Tian et al., 2015). This pathological process is crucial in forming a thrombus (Broos et al., 2011; Tian et al., 2015). As revealed by our study, complement and coagulation systems were activated in the acute and subacute phases of severe TBI, strengthening platelet aggregation due to possible C3-deficiency and the production of membrane attack complex (MAC) (Sauter et al., 2018; Chang, 2019; Fletcher-Sandersjöö et al., 2020). Thus, in addition to the individual function of complement and coagulation system or platelet activation, both pathways in synergy also play an essential role in the cerebrovascular circulation during the acute phase.

Consistent with TMT quantitative proteomics, we observed the significant upregulation of SPP1 and downregulation of SNCA and SYN1. Three proteins are highly related to neural activity. SNCA, an alpha-synuclein gene, is associated with synucleinopathies, a group of neurodegenerative diseases (Tagliafierro and Chiba-Falek, 2016). The binding of dopamine transporter (DAT 1) by SNCA induces the neurotransmission of dopamine (Butler et al., 2015). Intervention approaches for SNCA downregulation have been considered a feasible neuroprotective strategy for the last-onset of neurodegeneration (Tagliafierro and Chiba-Falek, 2016). SYN1 (synaptophysin I), a member of the family of phosphorylated proteins associated with synaptic vesicles, regulates synaptic transmission and plasticity (Fdez and Hilfiker, 2006). Downregulation of SYN1 has been proved to be a key factor for neuron structural defects and delayed synaptogenesis (Chin et al., 1995; Rosahl et al., 1995). SPP1 (also known as Osteopontin, OPN) is a secreted phosphoprotein 1 involved in synapse reorganization and improving functional recovery post-TBI (Chan et al., 2014; Powell et al., 2019; Zhou Y. et al., 2020). Gong et al. (2018) demonstrated the neuroprotective effects of SPP1 at day 3 post-acute intracerebral hemorrhage (ICH). Instead, another study noted that the mNSS did not show marked improvement using intranasal recombinant OPN (Jullienne et al., 2020). Thus, even though our research corroborated that SPP1 may be a neuroprotective biomarker, the most accurate route of administration needs further exploration.

The neurological decline is the dominant manifestation in the subacute stage compared with the acute phase. ALS is a neurodegenerative disease with loss of upper and lower motor neurons as pathological changes (Franz et al., 2019). In numerous epidemiological and clinical studies, the relationship between TBI and ALS remains controversial (Fournier et al., 2015; Franz et al., 2019). Nevertheless, the hippocampus has long been implicated in ALS, which explains why we discovered the ALS pathway. Pre-existing researches have shown chronic traumatic encephalopathy (CTE)- repetitive TBI- is a risk factor for ALS (Kiernan et al., 2015). In contrast, one-time acute focal injury caused by controlled cortical impact has not been associated with ALS onset (Thomsen et al., 2015). We demonstrate that longer onset time and severe trauma may also be risks factors for ALS besides a repetitive injury. Therefore, neuroprotective treatment should pay attention to the acute phase and the subacute stage. More remarkably, the subacute phase of severe TBI requires vigilance and attention in terms of ALS.

When we randomly selected and verified the VIM, GFAP, and S100A4 proteins, three were elevated in the subacute stage of severe TBI. GFAP, a class III intermediate filament protein specific to astrocytes, is a brain-specific biochemical marker (Bembea et al., 2011). It is associated with brain and neurological damage and up-regulated glial cells after central nervous system (CNS) injury (Panickar and Norenberg, 2005). VIM is an intermediate filament that maintains astrocyte integrity (Franke et al., 1982) and is over-expressed after CNS injury or neurodegenerative diseases (Pekny et al., 2007). These may be one of the molecular mechanisms of nerve injury during the subacute stage of TBI. S100A4 aggravates neuronal loss after brain injury and increases the damage of oxidized cells (Dmytriyeva et al., 2012). As a potentially critical factor for CNS injury, S100A4 exerts a neuroprotective effect and has also been used as a therapeutic target during the subacute phase of TBI (Dmytriyeva et al., 2012; Lipponen et al., 2016).

Some limitations exist in our present study. (1) This work provided only the proteomics clues to distinct mechanisms of acute and subacute stages of severe TBI. However, it offered potential signaling pathways and targets in future follow-up studies. (2) Some confounding factors, such as the bodyweight, sampling time, and conditions during sample transfer, should be eliminated as much as possible in the future. (3) We only obtained the hippocampus on the injured side of the CCI groups and the hippocampus on the same side of the Sham groups. However, it is necessary to conduct in-depth research on the contralateral brain tissue to further clarify the overall protein level changes in the future. (4) Technical duplicates make the experimental data more reproducible but fail to clarify the differences between the samples within the group.

## Conclusion

Overall, quantitative proteomic analysis coupled with bioinformatics revealed the dynamic pathophysiology across different stages of severe TBI. DEPs may be viewed as candidate targets of various periods. Apart from the platelet activation and nerve-related pathways, some abnormal secretions may also be pivotal in the acute phase of severe TBI. However, the significant enrichment of ALS alerts us to the persistence of nerve damage to the subacute phase, hinting to implement neuroprotective intervention at the earliest stage of severe TBI. It also indicates that the subacute phase does not necessarily decrease injury mechanisms. This work may facilitate the differential identification of biological processes involved in distinct stages of severe TBI.

## Data Availability Statement

The datasets presented in this study can be found in online repositories. The names of the repository/repositories and accession number(s) can be found in the article/Supplementary Material.

## Ethics Statement

The animal study was reviewed and approved by the Committee on the Use and Care of Animals of Central South University (approval number: 201603112).

## Author Contributions

YW: conceptualization, methodology, project administration, and supervision. WL: writing – original draft preparation and visualization. DZ, WL, and XG: software and investigation. DZ, WL, SW, and FH: data curation. YW, WZ, DZ, ZY, and WL: writing – review and editing. All authors: reviewed and approved the final manuscript.

## Conflict of Interest

The authors declare that the research was conducted in the absence of any commercial or financial relationships that could be construed as a potential conflict of interest.

## Publisher’s Note

All claims expressed in this article are solely those of the authors and do not necessarily represent those of their affiliated organizations, or those of the publisher, the editors and the reviewers. Any product that may be evaluated in this article, or claim that may be made by its manufacturer, is not guaranteed or endorsed by the publisher.

## Footnotes

1
https://metascape.org/

2
http://www.string-db.org/
