# Supplementary material for: Quantitative Proteomics Reveals the Dynamic Pathophysiology Across Different Stages in a Rat Model of Severe Traumatic Brain Injury
Source: Front Mol Neurosci. 2022 Jan 25;14:785938. doi: 10.3389/fnmol.2021.785938 (PMC8821658; doi:10.3389/fnmol.2021.785938)
Supplement: Supplementary file 1 [file Data_Sheet_1.zip › Supplementary Files/Supplementary File.docx]

**Supplementary**

**Quantitative Proteomics Reveals the Dynamic Pathophysiology across Different Stages in a Rat Model of Severe Traumatic Brain Injury**

Weikang Luo ^1,2#^, Zhaoyu Yang ^1,2#^, Wei Zhang ^3^, Dan Zhou ^4^, Xiaohang Guo ^5^, Shunshun Wang ^6^, Feng He ^7^, Yang Wang ^1,2^*

^1^ Institute of Integrative Medicine, Department of Integrated Chinese and Western Medicine, Xiangya Hospital, Central South University, Changsha, China

^2^ National Clinical Research Center for Geriatric Disorders，Xiangya Hospital, Central South University, Changsha, China

^3^ The College of Integrated Traditional Chinese and Western Medicine, Hunan University of Chinese Medicine, Changsha, China

^4^ Periodical office, Hunan University of Chinese Medicine, Changsha, China

^5^ Medical School, Hunan University of Chinese Medicine, Changsha, China

^6^ Postpartum Health Care Department, Hunan Provincial Maternal and Child Health Care Hospital, Changsha, China

^7^ Department of general surgery, Xiangya Hospital Central South University, Changsha, China


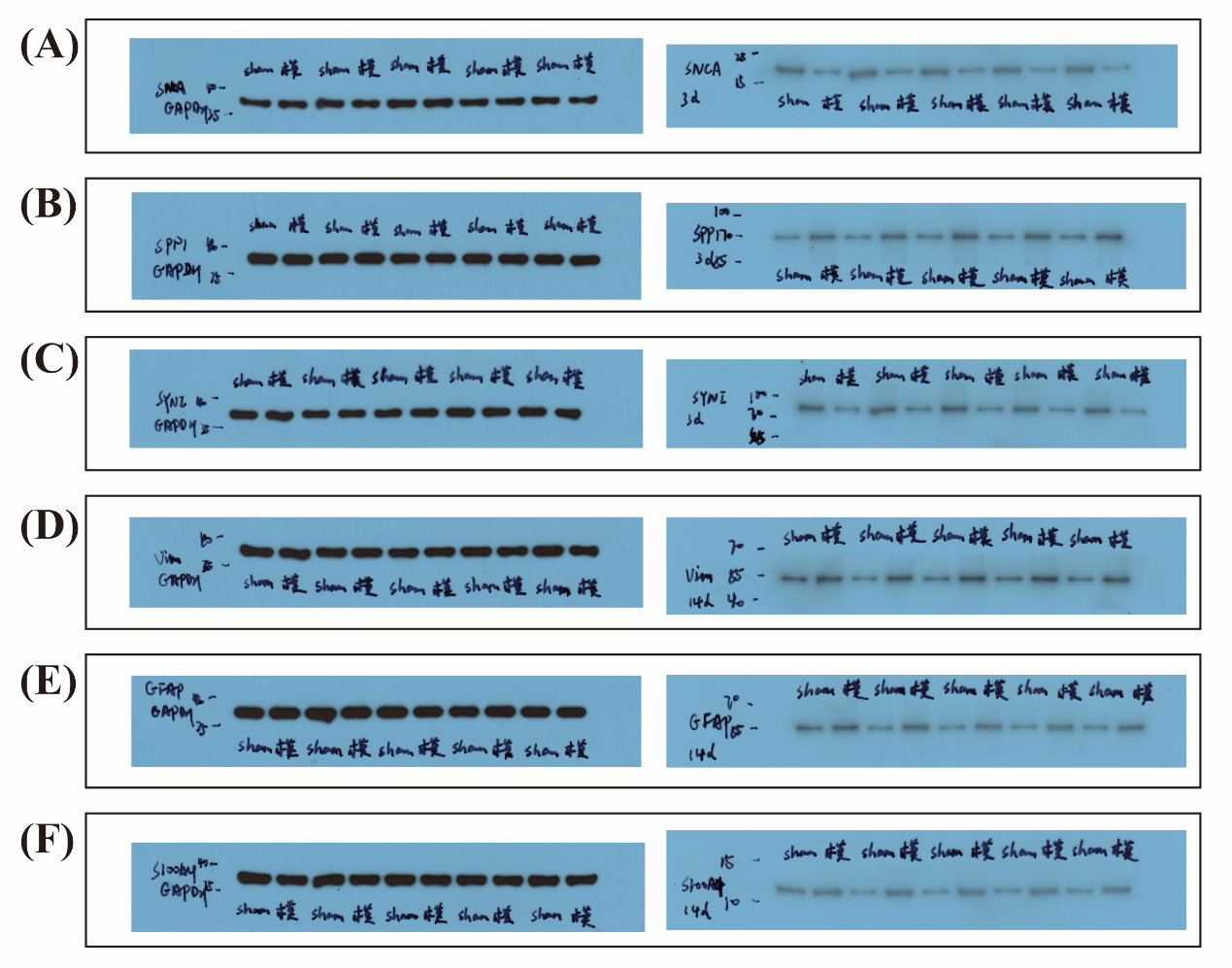


**Supplementary Figure 1. The western blot full gels.** (A) Protein expression and relative level of SNCA. Experiment repeated five times with similar results. (B) Protein expression and relative level of SPP1. Experiment repeated five times with similar results. (C) Protein expression and relative level of SYN1. Experiment repeated five times with similar results. (D) Protein expression and relative level of VIM. Experiment repeated five times with similar results. (E) Protein expression and relative level of GFAP. Experiment repeated five times with similar results. (F) Protein expression and relative level of S100A4. Experiment repeated five times with similar results.

**Table S1.** KEGG Pathway enrichment analysis of 312 DEPs in the acute phase of severe TBI.

| **Pathway** | **Enrichment** | **P-value** | **Count** | **LogP** |
| --- | --- | --- | --- | --- |
| Lysosome | 6.064295486 | 2.39825E-07 | 13 | -6.620104795 |
| Synaptic vesicle cycle | 8.735294118 | 8.05077E-07 | 9 | -6.094162686 |
| Complement and coagulation cascades | 6.525159461 | 9.61474E-06 | 9 | -5.017062313 |
| Platelet activation | 5.171415441 | 9.67081E-06 | 11 | -5.014537127 |
| Thyroid hormone synthesis | 6.68627451 | 2.52442E-05 | 8 | -4.597838802 |
| Gastric acid secretion | 6.418823529 | 3.40834E-05 | 8 | -4.467456698 |
| Regulation of actin cytoskeleton | 3.588505127 | 7.64212E-05 | 13 | -4.116786282 |
| ECM-receptor interaction | 5.66366782 | 8.42361E-05 | 8 | -4.074501807 |
| Keratan sulfate degradation | 30.08823529 | 8.75754E-05 | 3 | -4.057617717 |
| Insulin secretion | 5.597811218 | 9.15548E-05 | 8 | -4.038318902 |
| Focal adhesion | 3.574839837 | 0.000148881 | 12 | -3.827161605 |
| Other glycan degradation | 12.66873065 | 0.000237972 | 4 | -3.623473273 |
| Tuberculosis | 3.597506394 | 0.000264561 | 11 | -3.577474825 |
| Amoebiasis | 4.766453116 | 0.000281376 | 8 | -3.550712411 |
| Protein processing in endoplasmic reticulum | 3.647058824 | 0.000448699 | 10 | -3.348044655 |
| Glutamatergic synapse | 4.15010142 | 0.000714152 | 8 | -3.146209529 |
| Proteoglycans in cancer | 3.197783461 | 0.000715248 | 11 | -3.145543573 |
| Long-term potentiation | 5.470588235 | 0.000788963 | 6 | -3.102943253 |
| Amphetamine addiction | 5.470588235 | 0.000788963 | 6 | -3.102943253 |
| Oxytocin signaling pathway | 3.494117647 | 0.001162267 | 9 | -2.934693992 |
| Glycosphingolipid biosynthesis - ganglio series | 12.03529412 | 0.001782754 | 3 | -2.748908626 |
| Bacterial invasion of epithelial cells | 4.570364855 | 0.002017041 | 6 | -2.695285335 |
| Adrenergic signaling in cardiomyocytes | 3.366515837 | 0.002721823 | 8 | -2.565140198 |
| Hypertrophic cardiomyopathy | 4.247750865 | 0.002922052 | 6 | -2.534312071 |
| Cholinergic synapse | 3.69504644 | 0.002956958 | 7 | -2.529154822 |
| Rap1 signaling pathway | 2.785947712 | 0.003392505 | 10 | -2.469479519 |
| Endocytosis | 2.498676979 | 0.00339447 | 12 | -2.469227968 |
| Dilated cardiomyopathy | 4.011764706 | 0.003883602 | 6 | -2.410765333 |
| Glioma | 4.701286765 | 0.004200782 | 5 | -2.376669826 |
| Protein digestion and absorption | 3.882352941 | 0.004562788 | 6 | -2.340769752 |
| Vascular smooth muscle contraction | 3.397058824 | 0.004704525 | 7 | -2.327484229 |
| Glycosaminoglycan degradation | 8.596638655 | 0.004841156 | 3 | -2.315050961 |
| Dopaminergic synapse | 3.215536596 | 0.006326733 | 7 | -2.198820524 |
| Vitamin digestion and absorption | 7.522058824 | 0.007101946 | 3 | -2.148622607 |
| Tight junction | 2.865546218 | 0.007151002 | 8 | -2.145633116 |
| Amino sugar and nucleotide sugar metabolism | 5.014705882 | 0.008193408 | 4 | -2.086535444 |
| HIF-1 signaling pathway | 3.374381528 | 0.008932156 | 6 | -2.04904369 |
| Salivary secretion | 3.857466063 | 0.009646074 | 5 | -2.015649395 |
| MAPK signaling pathway | 2.323415853 | 0.011654535 | 10 | -1.933505067 |
| Amyotrophic lateral sclerosis | 4.298319328 | 0.013953545 | 4 | -1.855315444 |
| Staphylococcus aureus infection | 4.222910217 | 0.014815375 | 4 | -1.829287362 |
| Fc gamma R-mediated phagocytosis | 3.419117647 | 0.015671739 | 5 | -1.804882803 |
| Non-small cell lung cancer | 4.15010142 | 0.015710118 | 4 | -1.803820563 |
| ErbB signaling pathway | 3.380700595 | 0.016385842 | 5 | -1.78553124 |
| Glutathione metabolism | 3.882352941 | 0.019625279 | 4 | -1.707184162 |
| Phosphatidylinositol signaling system | 3.134191176 | 0.021994588 | 5 | -1.657684161 |
| Autophagy - animal | 2.735294118 | 0.02303969 | 6 | -1.637523373 |
| Melanogenesis | 3.008823529 | 0.025697759 | 5 | -1.590104745 |
| Renin secretion | 3.539792388 | 0.026539073 | 4 | -1.576114251 |
| Chemokine signaling pathway | 2.407058824 | 0.027248072 | 7 | -1.564664219 |
| AGE-RAGE signaling pathway in diabetic complications | 2.94982699 | 0.027690136 | 5 | -1.557674914 |
| Fat digestion and absorption | 4.513235294 | 0.028553703 | 3 | -1.544337559 |
| Retrograde endocannabinoid signaling | 2.893099548 | 0.029778102 | 5 | -1.52610299 |
| Apelin signaling pathway | 2.542667771 | 0.031457159 | 6 | -1.502280498 |
| Pertussis | 3.252782194 | 0.034743455 | 4 | -1.45912699 |
| Pathways in cancer | 1.814365947 | 0.034820898 | 12 | -1.458160029 |
| Inositol phosphate metabolism | 3.209411765 | 0.036238409 | 4 | -1.440830882 |
| Phospholipase D signaling pathway | 2.423213581 | 0.038408498 | 6 | -1.415572679 |
| Calcium signaling pathway | 2.22875817 | 0.038899711 | 7 | -1.410053622 |
| Oocyte meiosis | 2.66267569 | 0.040384808 | 5 | -1.393781975 |
| Leukocyte transendothelial migration | 2.593813387 | 0.044369103 | 5 | -1.352919352 |
| Proteasome | 3.761029412 | 0.045412461 | 3 | -1.342824966 |
| Cardiac muscle contraction | 2.97167756 | 0.045978408 | 4 | -1.337446068 |
| Sphingolipid metabolism | 3.684273709 | 0.047799744 | 3 | -1.320574426 |

**Table S2.** KEGG Pathway enrichment analysis of 76 DEPs in the subacute phase of severe TBI.

| **Pathway** | **Enrichment** | **P-value** | **Count** | **LogP** |
| --- | --- | --- | --- | --- |
| Amyotrophic lateral sclerosis (ALS) | 18.26785714 | 6.76709E-05 | 4 | -4.169598029 |
| Cardiac muscle contraction | 9.472222222 | 0.003931527 | 3 | -2.405438736 |
| Hypertrophic cardiomyopathy | 9.026470588 | 0.004500952 | 3 | -2.346695665 |
| Dilated cardiomyopathy | 8.525 | 0.005279693 | 3 | -2.27739137 |
| Adrenergic signaling in cardiomyocytes | 5.365384615 | 0.018521336 | 3 | -1.732327695 |

**Table S3.** KEGG Pathway enrichment analysis of 63 overlapping DEPs in the acute and subacute stages of TBI.

| **Pathway** | **Enrichment** | **P-value** | **Count** | **LogP** |
| --- | --- | --- | --- | --- |
| Complement and coagulation cascades | 17.89156627 | 8.71241E-06 | 5 | -5.05986158 |
| Pertussis | 16.05405405 | 0.000112371 | 4 | -3.949345756 |
| Proteoglycans in cancer | 7.173913043 | 0.000658019 | 5 | -3.181761853 |
| Staphylococcus aureus infection | 15.63157895 | 0.000934369 | 3 | -3.029481462 |
| B cell receptor signaling pathway | 12.54929577 | 0.001765121 | 3 | -2.753225463 |
| Leishmaniasis | 12.04054054 | 0.001987683 | 3 | -2.701652806 |
| NOD-like receptor signaling pathway | 7.071428571 | 0.002471072 | 4 | -2.607114521 |
| Salmonella infection | 10.73493976 | 0.002757512 | 3 | -2.559482634 |
| Natural killer cell mediated cytotoxicity | 9 | 0.004531391 | 3 | -2.343768436 |
| Focal adhesion | 5.881188119 | 0.004774064 | 4 | -2.321111732 |
| Amoebiasis | 8.821782178 | 0.004791702 | 3 | -2.319510194 |
| T cell receptor signaling pathway | 8.25 | 0.005773275 | 3 | -2.23857774 |
| Platelet activation | 6.9609375 | 0.009206377 | 3 | -2.03591123 |
| MAPK signaling pathway | 4.586872587 | 0.011273485 | 4 | -1.94794182 |
| JAK-STAT signaling pathway | 6.02027027 | 0.013617185 | 3 | -1.865912653 |
| Influenza A | 5.272189349 | 0.019344251 | 3 | -1.713448091 |
| Tuberculosis | 4.842391304 | 0.024135139 | 3 | -1.617350188 |
| Alzheimer disease | 4.842391304 | 0.024135139 | 3 | -1.617350188 |
| Epstein-Barr virus infection | 3.890829694 | 0.042006226 | 3 | -1.376686336 |

**Table S4.** Lists of proteins involved in three endocrine-related signal pathways.

| **Pathway** | **Up-regulated proteins** | **Down-regulated proteins** |
| --- | --- | --- |
| Thyroid hormone synthesis | Alb Gpx1 Ttr Slc5a5 Pdia4 | Prkcg Gsr Adcy9 |
| Gastric acid secretion | Slc4a2 Ezr | Camk2b Prkcg Camk2a Kcnj10 Calm2 Adcy9 |
| Insulin secretion |  | Camk2b Prkcg Snap25 Camk2a Rab3a Pclo Stx1a Adcy9 |

PROTEOMIC DATA AVAILABILITY STATEMENT

All proteomic data obtained have been deposited in the online data PRIDE Archive with the dataset identifier PXD029312 (http://www.ebi.ac.uk/pride/archive/login; Username: reviewer_pxd029312@ebi.ac.uk; Password: iMMMBwtS).
